# Supplementary figures and images for: Evaluating Propagation Techniques for Cannabis sativa L. Cultivation: A Comparative Analysis of Soilless Methods and Aeroponic Parameters
Source: Plants (Basel). 2024 Apr 30;13(9):1256. doi: 10.3390/plants13091256 (PMC11085668; doi:10.3390/plants13091256)

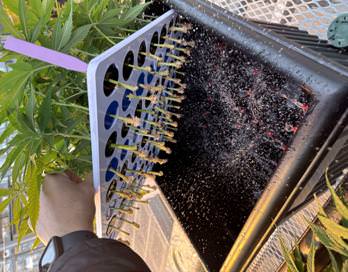

Supplement: Supplementary file 1 [file plants-13-01256-s001.zip › Figure_S1.thumbnail.jpg]

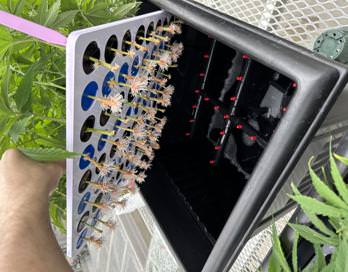

Supplement: Supplementary file 1 [file plants-13-01256-s001.zip › Figure_S2.thumbnail.jpg]

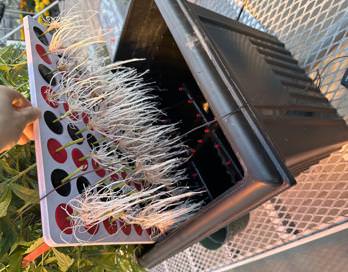

Supplement: Supplementary file 1 [file plants-13-01256-s001.zip › Figure_S3.thumbnail.jpg]

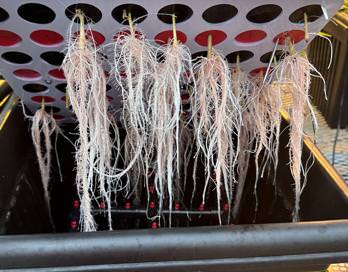

Supplement: Supplementary file 1 [file plants-13-01256-s001.zip › Figure_S4.thumbnail.jpg]

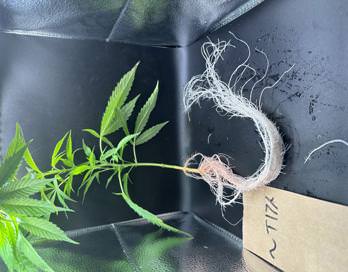

Supplement: Supplementary file 1 [file plants-13-01256-s001.zip › Figure_S5.thumbnail.jpg]

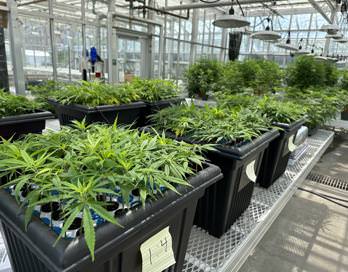

Supplement: Supplementary file 1 [file plants-13-01256-s001.zip › Figure_S6.thumbnail.jpg]

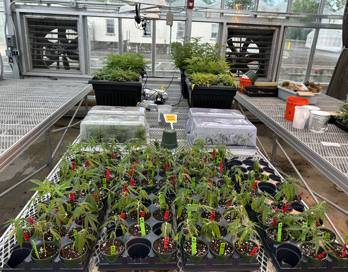

Supplement: Supplementary file 1 [file plants-13-01256-s001.zip › Figure_S7.thumbnail.jpg]

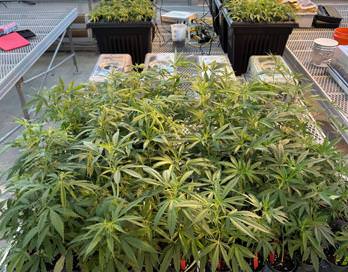

Supplement: Supplementary file 1 [file plants-13-01256-s001.zip › Figure_S8.thumbnail.jpg]

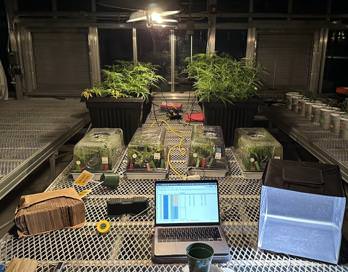

Supplement: Supplementary file 1 [file plants-13-01256-s001.zip › Figure_S9.thumbnail.jpg]
